# Vaccine-induced tumor regression requires a multi-step cooperation between T cells and myeloid cells at the tumor site

**DOI:** 10.1186/2051-1426-3-S2-P293

**Published:** 2015-11-04

**Authors:** Maxime Thoreau, Hwei Xian Leong Penny, Kar Wai Tan, Fabienne Regnier, Julia Miriam Weiss, Bernett Lee, Ludger Johannes, Estelle Dransart, Agnes Le bon, Jean-Pierre Abastado, Eric Tartour, Alain Trautmann, Nadege Bercovici

**Affiliations:** 1 Institut Cochin, Inserm U1016, CNRS UMR8104, Univ. Paris Descartes, Sorbonne Paris Cité, Equipe labellisée “Ligue contre le Cancer”, Paris, France; 2 Singapore Immunology Network, BMSI, A-STAR, Singapore, Singapore; 3 Institut Curie, INSERM U1143, CNRS UMR3666, Paris, France; 4 Inserm U970, PARCC, Université Paris Descartes, Sorbonne Paris Cité, Paris, France

## 

Most cancer immunotherapies under present investigation are based on the belief that cytotoxic T cells are the most important anti-tumoral immune cells, whereas intratumoral macrophages would rather play a protumoral role. We have challenged this antagonistic point of view and searched on the contrary for complementary contributions provided by tumor-infiltrating T cells and macrophages, reminiscent of those observed in anti-infectious responses. We demonstrate that, in a model of therapeutic vaccination, cooperation between myeloid cells and T cells is indeed required for tumor rejection. Vaccination elicited an early rise of CD11b^+^ myeloid cells that preceded and conditioned the intratumoral accumulation of CD8^+^ T cells. Conversely, CD8^+^ T cells and IFNg production activate myeloid cells and were required for tumor regression. A 4-fold reduction of CD8^+^ T cell infiltrate in CXCR3KO mice did not prevent tumor regression, whereas a reduction of tumor-infiltrating myeloid cells significantly interfered with vaccine efficiency. We show that macrophages from regressing tumors can eliminate tumor cells by TNFα release and phagocytosis. Altogether, our data suggest new strategies to improve the efficiency of cancer immunotherapies, by promoting intratumoral cooperation between macrophages and T cells.

